# Localization of SK2 channels relative to excitatory synaptic sites in the mouse developing Purkinje cells

**DOI:** 10.3389/fnana.2014.00154

**Published:** 2014-12-15

**Authors:** Carmen Ballesteros-Merino, José Martínez-Hernández, Carolina Aguado, Masahiko Watanabe, John P. Adelman, Rafael Luján

**Affiliations:** ^1^Instituto de Investigación en Discapacidades Neurológicas (IDINE), Departamento Ciencias Médicas, Facultad de Medicina, Universidad Castilla-La ManchaAlbacete, Albacete, Spain; ^2^Department of Anatomy, Hokkaido University School of MedicineSapporo, Japan; ^3^Vollum Institute, Oregon Health and Science UniversityPortland, OR, USA

**Keywords:** SK2 channels, cerebellar development, electron microscopy, immunohistochemistry, Purkinje Cells

## Abstract

Small-conductance, Ca^2+^-activated K^+^ (SK) channels regulate neuronal excitability in a variety of ways. To understand their roles in different neuronal subtypes it is important to determine their precise subcellular distribution. Here, we used biochemical, light microscopy immunohistochemical and immunoelectron microscopy techniques, combined with quantitative approaches, to reveal the expression and subcellular localization patterns of SK2 in the developing cerebellum. Using western blots, the SK2 protein showed a progressive increase during postnatal development. At the light microscopic level, SK2 immunoreactivity was very prominent in the developing Purkinje cells (PC), particularly in the molecular layer (ML). Electron microscopy revealed that throughout development SK2 was mostly detected at the extrasynaptic and perisynaptic plasma membrane of dendritic shafts and dendritic spines of PCs. However, there was some localization at axon terminals as well. Quantitative analyses and 3D reconstructions further revealed a progressive developmental change of SK2 on the surface of PCs from dendritic shafts to dendritic spines. Together, these results indicate that SK2 channels undergo dynamic spatial regulation during cerebellar development, and this process is associated with the formation and maturation of excitatory synaptic contacts to PCs.

## Introduction

The cerebellum plays a major role in fine motor control, contributes to memory, cognition, and emotional control (Ito, 2001, 2006). Purkinje cells (PCs) are central to those functions because they are the sole output neuronal population of the cerebellar cortex (Ito, 2001). The development of PCs and their elaborated synaptic contacts involves the interplay between gene expression and patterned activity driven by neurotransmitter receptors and ion channels (Altman and Bayer, 1997; Ben-Ari and Spitzer, 2010). Intracellular Ca^2+^ transients mediated by different types of Ca^2+^ channels are also important in both developing and adult PCs (Gruol et al., 1992; Eilers et al., 1996). Given the importance of the spatiotemporal patterns of Ca^2+^ transients and electrical activity in neuronal ontogenesis, small-conductance Ca^2+^-activated K^+^ (SK) channels may serve as feedback regulators of Ca^2+^-dependent processes during brain development.

In PCs, SK channels influence spike firing frequency, the modulation of Ca^2+^ transients in dendritic spines and they contribute to compartment-specific dendritic plasticity (Hosy et al., 2011; Ohtsuki et al., 2012). SK channels are voltage-independent and directly gated by variations in the concentration of intracellular Ca^2+^ and hence function as feedback modulators of neuronal activity (Köhler et al., 1996; Bond et al., 2005; Ngo-Anh et al., 2005; Luján et al., 2009). In PCs, P/Q-type Ca^2+^ channels that are essential for PC synaptogenesis provide the Ca^2+^ source for the activation of SK channels (Miyazaki et al., 2004, 2012). This functional coupling between P/Q-type and SK channels is highlighted by the findings that both of these channels have been implicated in cerebellar ataxia (Edgerton and Reinhart, 2003; Womack et al., 2004).

Three types of SK channel subunits, SK1-3 are widely expressed in the brain and they show overlapping yet distinct expression patterns (Stocker and Pedarzani, 2000; Sailer et al., 2002, 2004; Gymnopoulos et al., 2014). Immunoelectron microscopic studies proved the presence of SK2 and SK3 both at synaptic and extrasynaptic sites in the hippocampus and ventral tegmental area (Lin et al., 2008; Ballesteros-Merino et al., 2012, 2014; Soden et al., 2013). In the cerebellum, *in situ* hybridization studies indicate different cellular profiles of SK1-3 channels. Thus, while all three subtypes are expressed in granule cells, only SK2 is expressed in PCs (Gymnopoulos et al., 2014). During development, SK2 channels are also highly expressed in the cerebellum (Cingolani et al., 2002; Gymnopoulos et al., 2014), and in spite of their functional importance (Hosy et al., 2011; Ohtsuki et al., 2012), their involvement in cerebellar developmental processes and precise subcellular distribution is largely unknown. To determine the involvement of SK2 in cerebellar development and to understand how SK2 channel distribution develops relative to specific inputs, we used high-resolution immunoelectron microscopic techniques combined with quantitative analyses and 3-D reconstructions.

## Material and methods

### Tissue preparation

OF-1 mice from the day of birth (P0) to adulthood obtained from the Animal House Facility of the School of Medicine of the University of Castilla-La Mancha were used in this study for western blots and pre-embedding immunohistochemical analyses. The care and handling of animals prior to and during the experimental procedures was in accordance with Spanish (RD 1201/2005) and European Union regulations (86/609/EC), and the protocols were approved by the University’s Animal Care and Use Committee. For each developmental stage, the animals used were from different litters and were grouped as follows: P0, P5, P7, P10, P12, P15, P21 and P60, *n* = 3 per group for immunoblots; P0, P5, P10, P12, P15, P21 and P60, *n* = 3 per group for light microscopic immunohistochemistry; and P7, P12, P15, P21 and P60, *n* = 3 per group for electron microscopic immunohistochemistry.

For immunoblotting, postnatal P0 and P5 animals were deeply anesthetized by hypothermia, while P7 to P60 animals were deeply anesthetized by intraperitoneal injection of ketamine-xylazine 1:1 (0.1 ml/kg b.w.), before extraction of the brains, which were immediately quickly frozen. For immunohistochemical techniques, mice were anesthetized before transcardial perfusion with ice-cold fixative containing 4% paraformaldehyde, with or without 0.05% glutaraldehyde and 15% (v/v) saturated picric acid made up in 0.1 M phosphate buffer (PB, pH 7.4). Next, brains were removed from the skull and then immediately immersed in the same fixative for 2 h or overnight at 4°C. Tissue blocks containing the cerebellum were washed thoroughly in 0.1 M PB. Saggital 60 µm thick sections were cut on a Vibratome (Leica V1000, Wetzlar, Germany).

### Antibodies and chemicals

**Figure 1:**
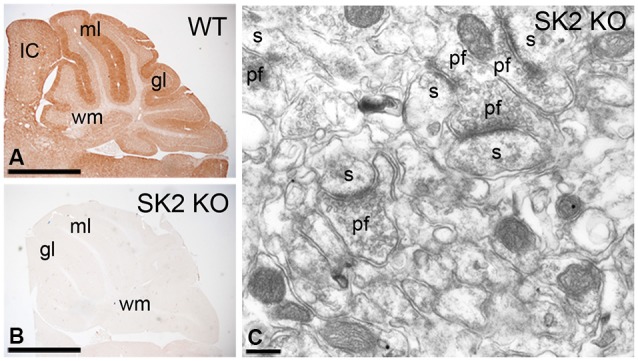
**Characterization of the affinity-purified anti-SK2 antibodies in the cerebellum of adult mice. (A,B)** Immunoreactivity for SK2 in the cerebellar cortex of (panel **A**) wild-type and (panel **B**) SK2 KO mice using a pre-embedding immunoperoxidase method at the light microscopic level. The pattern of SK2 immunoreactivity observed in the different layers of the cerebellar cortex in wild-type (WT) mice is completely missing in the SK2 KO mice. **(C)** Absence of SK2 immunolabeling in the cerebellum of SK2 KO mice using a pre-embedding immunogold method. ml, molecular layer; gl, granule cell layer; IC, inferior colliculus; s, spine; pf, parallel fiber terminal; wm, white matter. Scale bars: **A–B**, 1 mm; **C**, 0.2 µm.

Information on the characteristics and specificity of our antibodies against SK2 have been described previously (Cueni et al., 2008; Lin et al., 2008; Ballesteros-Merino et al., 2012). Briefly, affinity-purified rabbit antibodies to SK2 were raised against amino acid residues 536–574 of the mouse SK2 (accession number NM_080465). We have provided here further information on specificity in the cerebellum using immunohistochemical techniques (Figure 1). Indeed, to validate the specificity of the immunoreactions using different approaches, SK2 KO mice were used. At the light microscopic level, the pattern of immunoreactivity for SK2 observed in the cerebellar cortex of wild-type mice (Figure 1A) was completely missing in that of SK2 KO mice (Figure 1B). Furthermore, the complete lack of labeling in the SK2 KO mice at the electron microscopic level using the pre-embedding immunogold technique (Figure 1C) demonstrates that the immunolabeling is due to specific SK2 detection.

The monoclonal antibody against α-tubulin and the polyclonal raised in rabbit against Calbindin were obtained from Calbiochem (Germany) and Swant (Marly, Switzerland), respectively.

### Western blots

The cerebellum from different mice were homogenized in sucrose 320 mM, 2 mM EDTA, 10 mM HEPES, pH 7.4, and Protease Inhibitor Cocktail (Sigma-Aldrich) with a pestle motor (Sigma-Aldrich).The homogenized cerebellum was centrifuged at 5000 rpm at 4°C and the supernatant was ultracentrifuged at 25000 rpm at 4°C (Beckman Coulter Optima L-90 K Ultracentrifuge, CA, USA) using rotor SW40Ti (Beckman). Then, 100 µg of cerebellar membrane protein was prepared as Western blots and probed with anti-SK2 (1:400). Following application of a secondary antibody (goat anti-rabbit) coupled to horseradish peroxidase (1:3000), protein bands were visualized using the ECL blotting detection kit (SuperSignal West Dura, Pierce, Rockford, USA). Blot autoradiographs were quantified using densitometry analysis with a LAS4000 MINI (Fujifilm, Japan).

### Immunohistochemistry for light microscopy

Immunohistochemistry for light microscopy was performed using the immunoperoxidase method as described previously (Luján et al., 1996). Briefly, sections were incubated for 1 h in a blocking solution consisting of 10% normal goat serum (NGS) diluted in 50 mM Tris buffer (pH 7.4) and 0.9% NaCl (TBS), with 0.2% Triton X-100. Next, sections were incubated with the primary antibody (anti-SK2; 1–2 µg/ml diluted in TBS containing 1% NGS), followed by incubation in biotinylated goat anti-rabbit IgG (Vector Laboratories, Burlingame, CA, USA) secondary antibodies diluted in TBS with 2% NGS. Next, the sections were incubated in the avidin-biotin-peroxidase complex (ABC kit, Vector Laboratories). We revealed the bound peroxidase enzyme activity using 3, 3’-diaminobenzidine tetrahydrochloride (DAB; 0.05% in TB, pH 7.4) as the chromogen and 0.01% H_2_O_2_ as the substrate. Finally, after air-drying sections were coverslipped using a mounting medium before observation and analysis in a Leica photomicroscope (Leica DM 2500) equipped with a digital imaging camera (Leica DFC 500).

### Immunohistochemistry for electron microscopy

Immunohistochemistry for electron microscopy was performed using the pre-embedding immunogold technique as previously described (Luján et al., 1996). Briefly, free-floating sections were incubated in a blocking solution consisting of 10% NGS diluted in TBS. Next, sections were incubated with the primary antibody (anti-SK2; 1–2 µg/ml diluted in TBS containing 1% NGS), followed by incubation in the secondary antibody: goat anti-rabbit IgG coupled to 1.4 nm gold (Nanoprobes Inc., Stony Brook, NY, USA). Sections were postfixed in 1% glutaraldehyde and washed in double distilled water, followed by silver enhancement of the gold particles with a HQ Silver kit (Nanoprobes Inc.). Next, sections were treated with 1% osmium tetraoxide in 0.1 M PB and block-stained with uranyl acetate. These steps were followed by dehydration in graded series of ethanol and flat-embedding on glass slides in Durcupan (Fluka) resin. Regions of interest from the corresponding lobules of the cerebellum were cut at on an ultramicrotome (Reichert Ultracut E, Leica, Austria) and obtaining 70–90 nm thick-sections, and then they were collected on pioloform-coated single slot copper grids. Contrast for electron microscopy was performed on drops of 1% aqueous uranyl acetate and Reynolds’s lead citrate. Ultrastructural observation and analysis was carried out in a Jeol-1010 electron microscope. Electron photomicrographs were captured with ORIUS SC600B CCD camera (Gatan, Munich, Germany). Digitized images were then modified for brightness and contrast using Adobe PhotoShop CS5 (Mountain View, CA, USA) to optimize them for figure composition and quantitative analyses.

### Three-dimensional reconstructions

We used a similar procedure to that used previosly (Fajardo-Serrano et al., 2013). The three-dimensional (3D) reconstruction of SK2-immunopositive PC dendritic spines and shafts was carried out using 18–25 serial ultrathin sections obtained from pre-embedding immunoreacted tissue at four different postnatal ages: P7, P12, P15 and P21. SK2 labeled structures during postnatal development were classified based on unambiguous ultrastructural criteria. PC dendrites were identified based on the presence of subsurface membrane cisterns and emerging spines from the dendritic shaft. PC spines were identified as small profiles showing membrane continuity with the dendritic shaft, the presence of a prominent postsynaptic density and their opposition to parallel fiber axon terminals, recognized by their small size, a few mitochondria and large concentration of tightly packed, round synaptic vesicles. Synaptic contacts were identified as parallel membranes separated by widened clefts and associated with membrane specializations. Those synapses that displayed a prominent density on the postsynaptic element and established on dendritic spines were classified as asymmetrical or excitatory, while those lacking such a prominent density and established on dendritic shafts were classified as symmetrical or inhibitory.

Tissue areas from the very surface (<5 µm) and containing the molecular layer (ML) of the cerebellar cortex were randomly selected and captured at a final magnification of 50,000X. Then, serially sectioned images were imported into the Reconstruct software (Fiala, 2005) and the image stacks were aligned. Next, the plasma membrane of each dendrite, spine and spine head was traced manually. We obtained a first section of the series from the surface of the tissue, and then we measured the density of immunoparticles for SK2 along the plasma membrane of PCs in all sections used for 3D reconstruction, in order to determine whether the signal intensity was similar throughout the depth of the series, was measured. This analysis showed the lack of significant changes in immunoparticle density within the series of serial sections.

### Quantitative analyses

To determine the relative frequency of SK2 immunoreactivity in PCs, we employed 60-µm saggital slices processed for pre-embedding immunogold immunohistochemistry, using procedures similar to those used previously (Luján et al., 1996; Luján and Shigemoto, 2006). Briefly, for each developmental age (P7, P12, P15, P21 and P60), three tissue samples were obtained from each animal, making a total of *n* = nine blocks per age. Because immunoreactivity decreases with depth in the pre-embedding reactions, electron microscopic serial ultrathin sections were cut close to the surface of each block, thus mimimising false negatives. We defined areas with optimal gold labeling at approximately 5–10 µm from the cutting surface and estimated the quality of immunolabeling within those areas. Then, we photographed randomly selected areas from the selected ultrathin sections and captured images at a final magnification of 45,000X. Quantification of immunogold labeling was carried out in reference areas totaling ~2,000 µm^2^ for each age. Quantification of immunolabeling was performed in two different ways.

#### Percentage of immunoparticles

To determine the frequency of SK2, immunoparticles identified in each reference area and present in different subcellular compartments (dendritic spines, dendritic shafts, and axon terminals) were counted. Data were expressed as percentage of immunoparticles in each subcellular compartment (Table 1).

**Table 1:** **Summary of immunogold labeling for extrasynaptic SK2 during postnatal development**.

	Plasma membrane number / %	Intracellular number / %	Total immunogold number / %
**P7**
*Dendritic spines*	88/7,6	107/9,2	195/16,9
*Dendritic shafts*	415/35,9	521/45	936/80,9
*Axon terminals*	19/1,6	7/0,6	26/2.2
**P12**
*Dendritic spines*	191/10,8	237/13,4	428/24,1
*Dendritic shafts*	698/39,2	581/32,7	1279/72,1
*Axon terminals*	55/3,1	13/0,7	68/3,8
**P15**
*Dendritic spines*	588/30,2	157/8,1	745/38,3
*Dendritic shafts*	541/27,9	434/22,3	975/50,1
*Axon terminals*	176/9	51/2,6	227/11,7
**P21**
*Dendritic spines*	697/32,3	213/9,9	910/42,1
*Dendritic shafts*	602/27,9	422/19,6	1024/47,4
*Axon terminals*	172/8	55/2,5	227/10,5
**P60**
*Dendritic spines*	798/33,4	241/10,1	1039/43,4
*Dendritic shafts*	639/26,7	415/17,3	1054/44,1
*Axon terminals*	232/9,7	67/2,8	299/12,5

#### Distribution of immunoparticles in dendritic spines

We established the abundance of SK2 channel immunoreactivity in PC dendritic spines relative to neurotransmitter release sites during development at P7, P12, P15 and P21. We counted immunoparticles recognized in each reference area and present in PC dendritic spines. Differences in the distribution of immunoparticles among different samples were not statistically significant (*P* = 0.64, Kolmogorov-Smirnov non-parametric test) and, therefore, we pooled the data. Next, the length of the PC dendritic spine membrane was measured from the edge of the postsynaptic density, as well as the position of the center of each immunoparticle associated to the plasma membrane (*n* = 156 for P7; *n* = 291 for P12; *n* = 387 for P15; and *n* = 452 for P21), using a digitizing tablet and appropriate software (Image J). To obtain a normalized value of the abundance of SK2 channels during postnatal development along the dendritic spine, the number of immunoparticles was expressed as relative frequency in 60-nm bins.

### Controls

To test method specificity in the procedures for both light and electron microscopy, the antibody anti-SK2 was tested on cerebellar sections obtained from the SK2 knockout mice (Figure 1). Following immunohistochemical procedures, the labeling disappeared completely in areas where a strong signal was present in sections from wild-type animals (Figure 1). Furthermore, the primary antibody was either omitted or replaced with 5% (v/v) normal serum of the species of the primary antibody. Under these conditions, no selective labeling was observed. Staining patterns were also compared to those obtained using an antibody anti-Calbindin, and only the antibody anti-SK2 consistently labeled the plasma membrane.

### Data analysis

Statistical analyses for morphological data were performed using SigmaStat Pro (Jandel Scientific) and data were presented as mean ± SEM. “n” indicates number of animals. We used ANOVA followed by the Bonferroni test for multiple comparisons to determine statistical significance, defined as *P* < 0.05. For the data obtained using electron microscopy, statistical significance in the distribution of immunoparticles among samples was assessed with the Kolmogorov–Smirnov non-parametric test.

## Results

### Developmental profile of SK2 protein in the cerebellum

**Figure 2:**
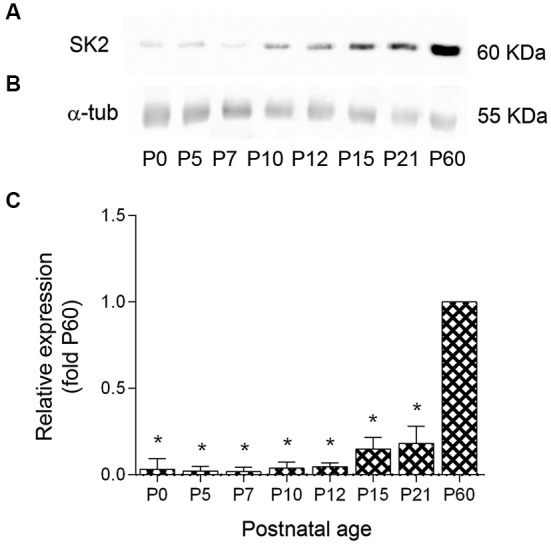
**Expression profile and quantitative immunoblot analysis of SK2 protein expression in mouse cerebellum during postnatal development. (A)** The western blots measurements from three animals were averaged to compare the protein level for each age. The SK2 antibody recognized a band of ~60 kDa at all ages. **(B)** The band for α-tubulin was used as a control to normalize SK2 expression at different developmental ages. **(C)** During postnatal development, SK2 protein was detected at birth, increased 2-fold between P0 and P7, increased 2-fold between P7 and P10–12, increased 3–4 times between P12 and P15–P21 and then increased 5-fold between P21 and P60, reaching the highest expression levels. Densitometry measurements from nine independent experiments were averaged for each developmental age and normalized to P60. Error bars indicate SEM. ^*^p < 0.05.

To determine the profile of SK2 channel expression during postnatal development Western blots were prepared with membrane protein fractions derived from mice cerebellum of different postnatal ages. The SK2 antibody gave a band of ~60 KDa at all developmental age analyzed (Figure 2A), consistent with the size of the SK2 protein (Strassmaier et al., 2005). SK2 expression increased after birth, but was low during the first and second postnatal week and showed the highest expression level at P60 (Figures 2A,C). We normalized the SK2 expression at different developmental ages of the cerebellar cortex by using the band for α-tubulin as a control (Figure 2B). Densitometry measurements from six different experiments were averaged at each postnatal age, revealing a 31-fold increase in SK2 protein expression between ages P0 and P60, a 2-fold increase in protein expression between ages P7 and P10-P12, and a 3-fold increase between P12 and P21. In the adult (P60), the expression of SK2 was 5 to 6-fold higher than that detected at P15-P21 (Figure 2C).

### Maturation of SK2 expression in the developing Purkinje cells

**Figure 3:**
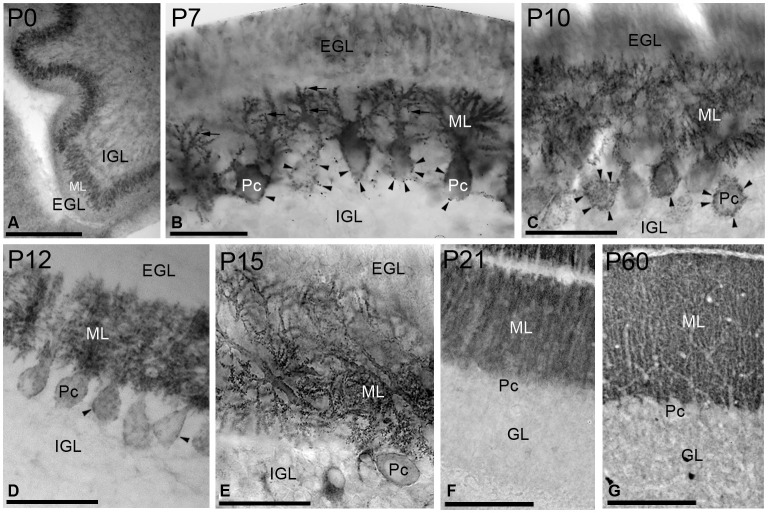
**Immunoreactivity for SK2 in the cerebellar cortex during postnatal development using a pre-embedding immunoperoxidase method. (A)** At P0, strong immunolabeling for SK2 was found in the undifferentiated PC and molecular (ML) layers. **(B)** At P7, immunoreactivity for SK2 concentrated in PC somatic spines (arrowheads) and in spine-like structures in the growing dendritic trees in the ML (arrows). Staining in the external (EGL) and internal (IGL) granular layers was very weak for SK2. **(C,D)** At P10–P12, SK2 concentrated in PC dendrites and spines in the ML, but still very prominent in somatic spines (arrowheads). **(E)** At P15, SK2 disappeared completely from the somata and a strong punctate labeling, corresponding to PC spines, was found in the ML. **(F,G)** At P21, the pattern and intensity of SK2 immunoreactivity closely matched that of the adult (P60, *panel* ***G***). The highest intensity of SK2 immunolabeling was observed in the ML, whereas the granule cell layer (GL) showed a uniformly low intensity of SK2 immunoreactivity. Pc, Purkinje cell. Scale bars: (**A**,**G**) 0.2 µm; F, 0.1 µm; (**B**) (**C–E**) 0.05 µm.

Next, we performed immunohistochemical techniques at the light microscopic level on sagittal sections through the vermis of the cerebellar cortex to study the expression and distribution of SK2 during postnatal development (Figure 3). To eliminate variations due to regional differences and maturation of the different lobules, the descriptions of our results were undertaken using the same lobules (IV–V) of the cerebellar cortex.

At birth (P0), intense immunoreactivity for SK2 was mainly observed in the undifferentiated PC layer, whereas very weak labeling was found in the ML (Figure 3A). At P7, SK2 immunoreactivity was detected throughout all branches of the PC dendritic tree emerging from cell bodies (Figure 3B). Strong punctuate labeling was observed around the somata of PCs, corresponding to the SK2 distribution in somatic spines, as well as some spines on the growing dendritic tree (Figure 3B). A moderate to weak SK2 immunoreactivity was observed in the external and internal granular layers (Figure 3B). At P10, strong SK2 labeling was confined to PCs in somatic spines in the PC layer and PC dendrites in the ML, with weaker SK2 immunoreactivity in granule cells (Figure 3C). Two days later, at P12, SK2 expression spread progressively throughout the entire dendritic arbor and SK2-expressing somatic spines disappeared from the basal portion of the PC somata and only remained to the upper part of the PC cell bodies (Figure 3D).

At P15, only weak SK2 immunoreactivity outlined PC somata but strong and punctuate immunolabeling was observed in the ML (Figure 3E). At P21, SK2 immunoreactivity was mostly detected in the ML (Figure 3F). At this age, the pattern and intensity of SK2 expression was very similar that seen in the adult (P60), characterized by a strong immunolabeling in the ML and faintly stained PC somata, and a much weaker labeling in the granule cell layer (Figure 3G).

### Subcellular localization of SK2 during postnatal development

To resolve the subcellular distribution and the acquisition of SK2 during development, dendritic shafts and spines of PCs were 3D-reconstructed from serial ultrathin sections labeled for SK2 at four representative stages: P7, before the main period of PF synapse formation along PC dendrites; P12, when most PF synapses have already formed; P15, when the final maturation of PF synapses begins; and P21, when the maturation of PF synapses attains adulthood characteristics.

**Figure 4:**
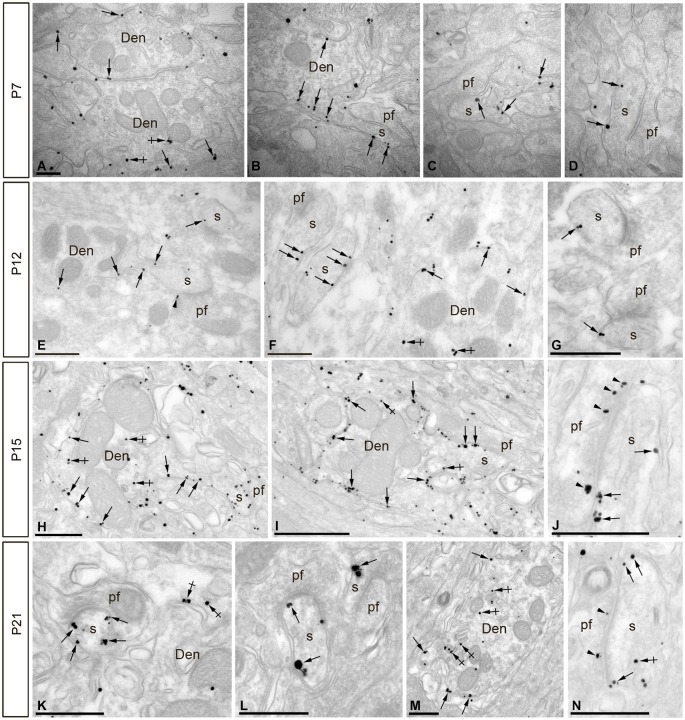
**Electron micrographs of the cerebellar cortex showing immunoreactivity for SK2 during postnatal development as detected using a pre-embedding immunogold method**. **(A–D)** At P7, immunoparticles for SK2 were mainly located at the extrasynaptic plasma membrane of PC dendritic shafts (Den; arrows) and at intracellular sites in such compartments (crossed arrows). To a lesser extent, SK2 immunoparticles were also detected along the extrasynaptic plasma membrane of PC dendritic spines (s; arrows) establishing asymmetrical synapses with parallel fibers (pf), and mostly far from perisynaptic sites. **(E–G)** At P12, immunoreactivity for SK2 increased progressively at PC spines (s) compared to labeling in dendritic shafts (Den) in the ML. SK2 immunoparticles were observed at extrasynaptic sites (arrows) in spines (s) establishing asymmetrical synapses with parallel fibers (pf). **(H–J)** At P15, an increase in SK2 immunoparticles was detected in extrasynaptic plasma membrane (arrows) of both PC dendritic shafts (Den) and spines (s). **(K–N)** At P21, the highest density of immunoparticles for SK2 was observed on the extrasynaptic and perisynaptic (arrows) plasma membrane of PC dendritic spines (s) establishing synapses with parallel fibers (pf). At all ages studied, SK2 immunoparticles were also found in the active zone (arrowheads) of parallel fiber terminals (pf), and examples are illustrated in panels **J,N**. Scale bars: **A–D**, 0.2 µm; **E–N**, 0.5 µm.

At P7, the majority of SK2 immunoparticles were found in dendritic shafts and immature dendritic spines, both along the plasma membrane and at intracellular sites of PCs (Figures 4A–D; Table 1). Although a few PF terminals were labeled for SK2 at the presynaptic level, they were rare at this developmental age (Table 1). At P12, immunoreactivity SK2 was primarily found in dendritic shafts and dendritic spines, and to a lesser extent in PF axon terminals establishing asymmetrical synapses with PCs (Figures 4E–G; Table 1). Most SK2 immunoparticles were detected along the plasma membrane of PC dendritic shafts and dendritic spines (Figures 4E–G; Table 1). At P15, the majority of SK2 immunoparticles were observed at the plasma membrane of dendritic shafts and dendritic spines, and associated with intracellular sites of PCs (Figures 4H–I; Table 1). Furthermore, SK2 immunolabeling was frequently seen in PF axon terminals (Figure 4J; Table 1). At P21, the highest density of SK2 immunoparticles was detected along the extrasynaptic plasma membrane of PC dendritic spines, followed by dendritic shafts (Figures 4K–L; Table 1). At the presynaptic level, immunolabelling for SK2 was identifiable in PF synapses and their terminals (Figures 4M–N; Table 1). These distribution patterns closely matched those observed in the adult (P60) animal (Table 1).

### Developmental distribution of SK2 in PCs relative to excitatory synapses

**Figure 5:**
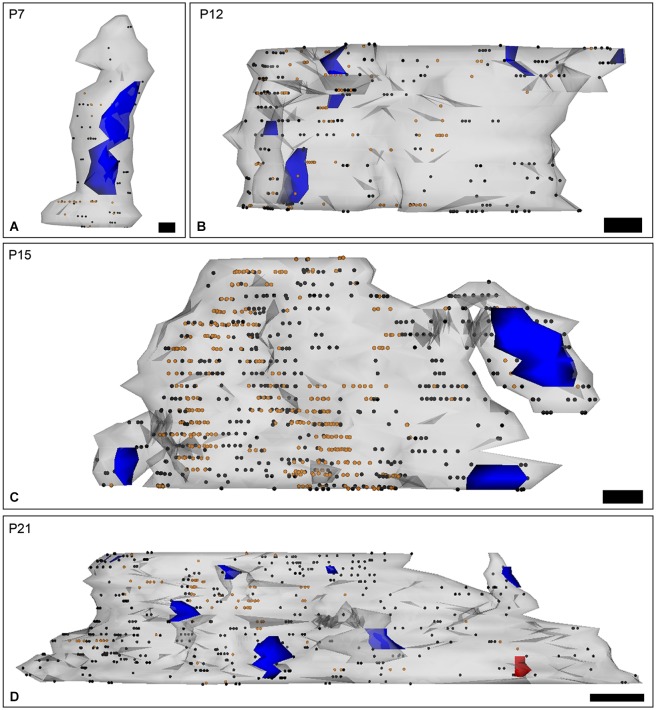
**Three-dimensional reconstruction of PC dendrites with spines labeled for SK2 channels during postnatal development. (A–D)** The reconstructions were carried out from serial ultrathin sections (*n* = 30) and show the localization of SK2 in relation to excitatory synaptic junctions (blue areas). Black dots represent immunogold particles on the front surface and orange dots show immunogold particles at intracellular sites. The red area is an inhibitory synapse. **(A)** At P7, SK2 immunoparticles were randomly distributed along PC dendritic shafts. **(B)** At P12, SK2 immunoreactivity was mainly found at extrasynaptic sites throughout PC dendrites, and observed an increasing association of SK2 with PF synapses on PC spines. **(C)** At P15, immunoparticles for SK2 were found forming clusters along PC dendritic shafts and close to PF synapses on PC spines. **(D)** At P21, however, more SK2 immunoparticles were located in PC spines, particularly around synaptic contacts with PF inputs, than at previous ages. Scale bars: P7, 100 nm length and 70 nm high, P12–P15, 200 nm length and 70 nm high; P21, 500 nm length and 70 nm high.

To determine the spatial relationship of SK2 on developing PCs relative to excitatory synapses, 3D reconstructions were carried out from serial ultrathin sections (Figures 5A–D). At P7, immunoparticles were not associated with or close to PF synapses, but instead they distributed in a random manner along dendritic shafts of PCs (Figure 5A). At P12, an increase in SK2 immunolabeling was observed at extrasynaptic sites along PC dendrites, parallel to a progressive association with PF synapses established on PC spines (Figure 5B). At P15, immunoparticles for SK2 were detected forming clusters along PC dendritic shafts, and in some occasions, they were close to PF synapses established on PC spines (Figure 5C). At P21, most of the SK2 immunoparticles were distributed in dendritic spines of PCs, specially around PF synaptic contacts, as well as clustered along PC dendritic shafts (Figure 5D).

**Figure 6:**
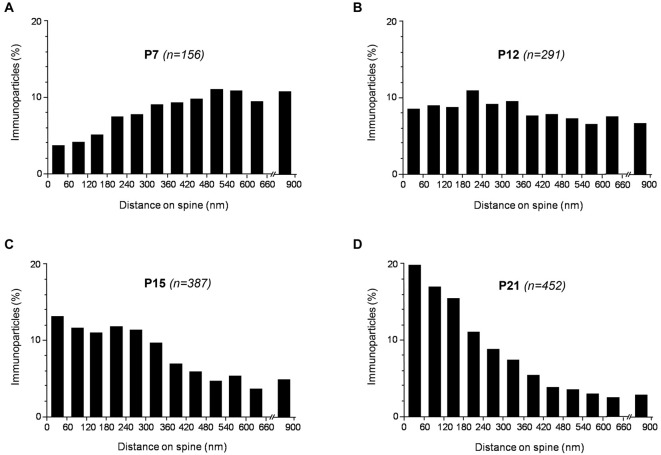
**Distribution of SK2 immunoreactivity relative to glutamate release sites in PC dendritic spines, as assessed from pre-embedding immunogold reactions, at P7 (A), P12 (B), P15 (C) and P21 (D)**. Immunoparticles were recorded in 60-nm-wide bins along the extrasynaptic plasma membrane of dendritic spines. Data are expressed as the proportion of immunoparticles at a given distance from the edge of the synaptic specialization. At P7, SK2 was more concentrated at some distance from excitatory synapse, at P10 it was uniformly distributed along the dendritic spine plasma membrane, while at P21 it was more associated with excitatory synapses. Overall, the measurements show that SK2 progressively accumulates around excitatory synapses established by PFs on PC spines during postnatal development.

These results were extended by determining the distribution of SK2 immunolabeling relative to glutamate release sites, measured as the number of immunoparticles along the plasma membrane of PC dendritic spines. Immunoparticle counts were then normalized to relative frequency in 60-nm bins (Figure 6). During postnatal development, immunoparticle density increased along the dendritic spine plasma membrane. Thus, 28% of all immunoparticles at P7, 46% at P10, 59% at P15 and 72% at P21, were distributed in the 300 nm wide band around the PC-PF synapse (Figures 6A–D). The developmental changes in the subcellular localization of SK2 in relation to the neurotransmitter release site shown here, combined with the 3-D reconstruction of PC dendrites and spines, confirm the progressive association of SK2 with PF-PC excitatory synapses during postnatal development.

## Discussion

A major factor affecting the response of a neuron during synaptic transmission is the distribution of ion channels along the surface of the neuron. Their distribution patterns are established during the process of synaptogenesis and may be dynamically modified during maturation of synaptic contacts (Luján et al., 2005). PCs are ideal neurons to study distribution patterns of ion channels, because they receive two distinct types of glutamatergic synapses (PF and CF) that which undergo changes in architecture during development (Landis, 1987; Landis et al., 1989). In the present study, we determined the expression, cellular and subcellular locations in which SK2 channels perform their cerebellar functions during development, with particular emphasis to the postnatal development of PCs.

### Light microscopic distribution of SK2 channels in the developing cerebellum

The important variations in Ca^2+^ conductances taking place during brain development indicate that underlying changes also take place in the expression of Ca^2+^ channels and, consequently Ca^2+^ activated channels such as SK channels. In the cerebellum, we observed that SK2 is widely distributed during postnatal development, particularly in the ML of the cerebellar cortex, where the strongest immunolabeling was observed. Our data also show pronounced differences in SK2 protein levels between P0 and P60. The SK2 expression increases progressively during the first, second and third weeks of postnatal development, reaching high, stable levels in adult cerebellum. This protein expression pattern is consistent with *in situ* hybridization and electrophysiological reports (Hosy et al., 2011; Gymnopoulos et al., 2014), but not as congruous with previous studies performed in rats showing that SK2 mRNA and protein had high expression at birth and then declined during the first 3 weeks of postnatal development (Cingolani et al., 2002). The explanation for this discrepancy is not clear but likely reflects a species difference. The results presented here rely on SK2 antibodies that have been validated using tissue from SK2 knockout mouse brain, engendering confidence in the results. Our data are further supported by electrophysiological studies showing that apamin increased the spike firing rate of PCs obtained from adult rats to a similar degree as observed in young rats (Womack and Khodakhah, 2003; Hosy et al., 2011), suggesting that SK2 channel function is not down-regulated in adult rat PCs. It would be interesting to examine in the future the possible interspecies differences related with the SK2 localization in specific cerebellar cell types.

The three SK channel subunits (known as SK1, SK2, and SK3) are expressed in the cerebellum (Stocker and Pedarzani, 2000; Sailer et al., 2002, 2004; Bond et al., 2004) but have distinct cellular expression patterns. The three channel subunits are expressed in granule cells, but only SK2 is expressed in PCs and SK3 in Golgi cells, and during development, neither SK1 nor SK3 are expressed in differentiating or developing PCs (Cingolani et al., 2002; Gymnopoulos et al., 2014). It has been further suggested that apamin sensitivity in PCs could be exclusively assigned to SK2 channels (Grunnet et al., 2001). Consistent with these data, we found that the SK2 protein was widely distributed in developing and adult PCs and the immunolabeling was particularly strong in their dendritic tree. We also found a weak immunoreactivity in the granule cell layer, mainly ascribable to the staining of granule cells. Altogether, these data strongly support the hypothesis that the apamin sensitive currents in PCs reflect homotetrameric SK2 channels at all postnatal developmental stages.

### Association of SK2 with excitatory synapses in the developing PCs

The location and density of SK channels in specific subcellular compartments heavily influence the functional consequences for their activation. Previous studies of SK2 expression have shown that SK2 is present postsynaptically in pyramidal cells of the hippocampus and dopaminergic cells of the substantia nigra and ventral tegmental area (Lin et al., 2008; Ballesteros-Merino et al., 2012; Deignan et al., 2012; Soden et al., 2013). Here we describe the first quantitative high-resolution information showing that SK2 is mainly located along the extrasynaptic plasma membrane of dendritic shafts and in spines of developing and adult PCs in the cerebellum. These emphasize the prominent role of SK2 channels in dendrites and spines observed in electrophysiological studies (Cingolani et al., 2002; Womack and Khodakhah, 2003; Belmeguenai et al., 2010; Hosy et al., 2011; Ohtsuki et al., 2012). In addition to the strong labeling detected at dendrites and spines, a low but consistent axon terminal immunoreactivity for SK2 has been observed. Similar data was previously described for SK2 in the hippocampus (Ballesteros-Merino et al., 2012). Although the function of SK2 channels at presumed presynaptic compartments is still unknown, their proximity to the axonal active zones strongly indicates that they may influence neurotransmitter release, perhaps through functional coupling to P/Q-type Ca^2+^ channels that drive release.

The preferential dendritic localization of SK2 permits SK2 channel activity to influence different aspects of neuronal physiology. For example in CA1 pyramidal neurons, SK2 channels in spines are activated by Ca^2+^ influx through closely positioned NMDARs within the postsynaptic membrane to modulate synaptic responses. A different population of SK2 channels in the dendrites is activated following the Ca^2+^ influx occurring after the opening of voltage-gated Ca^2+^ channel and influence dendritic excitability (Cai et al., 2004). In thalamic nRt neurons, dendritic SK2 channels, fueled by Ca^2+^ influx through T-type Ca channels compete with SERCA pumps for these Ca^2+^ ions to shape spike patterning. SK2 channels are also fueled by ryanodine-sensitive and IP_3_-sensitive stores via G protein-coupled receptors (Adelman et al., 2012), including group I mGlu receptors (García-Negredo et al., 2014).

One of the most interesting features coming out from the present data about the expression and distribution of SK2 during postnatal development is their clear change in subcellular localization that take place on the neuronal surface of PCs, which is a parallel process to the formation and maturation of excitatory synapses. Parallel and climbing fibers, the two excitatory inputs received by PCs, develop during the first and second postnatal weeks (Altman and Bayer, 1997). Soon after birth, the development of climbing fibers takes place and establishes functional excitatory synapses on the cell bodies of PCs. SK2 immunoreactivity was present in PC somatic spines, suggesting that SK2 channels may also mediate responses following the stimulation of climbing fiber during development. On the other hand, the establishment of parallel fiber synapses on dendritic spines of PCs takes place by P7, although the major period of parallel fiber synapse formation occurs at P10 (Altman and Bayer, 1997). It is interesting to mention that at P7 we only detected a low density of SK2 immunoparticles, which were randomly distributed, while at P12 the number of SK2 immunoparticles increased on the surface of dendritic spines and shafts of PCs. Parallel to maturation of cerebellar synapses we found clustering of SK2 channels along PC dendritic shafts and dendritic spines at P15, and the main presence of SK2 channels in PC spines at P21. A closer investigation at how SK2 channels develop relative to neurotransmitter release sites in PC spines showed a progressive accumulation to perisynaptic positions. The change in the subcellular localization of SK2 detected during developmental in this study is the first example of the lateral movement experienced by an ion channel that is localized in a place different from its final destination, the dendritic spines and associated excitatory synapse, in a process that is reached during synapse maturation. Similarly, this type of dynamic regulation during postnatal development has also been described for the mGlu1α and GABA_B_ receptors in PCs (López-Bendito et al., 2001; Luján and Shigemoto, 2006). Other than the expected role in synaptic neurotransmission, the dynamic regulation of SK2 in PCs during postnatal development is likely connected with plasticity processes taking place in PCs. Interestingly, recent electrophysiological data in PCs have shown that a form of intrinsic plasticity mediated by SK channel down-regulation is associated with an enhancement of Ca^2+^ transients in spines. Furthermore, such increased Ca^2+^ signaling lead to a lower probability for the induction of LTP (Belmeguenai et al., 2010; Hosy et al., 2011). In addition, activity-dependent plasticity of dendritic intrinsic excitability needs SK2 channel regulation and enables PCs to locally adjust dendritic processing properties (Ohtsuki et al., 2012). In PCs, an increase in the function SK channel activity has been shown to improve ataxia (Walter et al., 2006). These observations suggest that PCs might use activity-dependent excitability mediated by SK2 channels to tune the electrical output of the cerebellar cortex.

## Conflict of interest statement

The authors declare that the research was conducted in the absence of any commercial or financial relationships that could be construed as a potential conflict of interest.
